# Diagnostic Value of Non-invasive Scoring Systems in the Prediction of Esophageal Varices in Patients with Liver Cirrhosis—Single Center Experience

**DOI:** 10.3390/medicina58020158

**Published:** 2022-01-20

**Authors:** Tijana Glisic, Milica Stojkovic Lalosevic, Tamara Milovanovic, Ivan Rankovic, Marija Stojanovic, Aleksandar Toplicanin, Marko Aleksic, Vladimir Milivojevic, Jelena Martinov Nestorov, Iva Lolic, Dusan D. Popovic

**Affiliations:** 1 Clinic of Gastroenterology and Hepatology, Clinical Center of Serbia, 11000 Belgrade, Serbia; tamara.alempijevic@med.bg.ac.rs (T.M.); doctorranke@gmail.com (I.R.); aleksandartoplicanin4@gmail.com (A.T.); dotorevlada@gmail.com (V.M.); jelenamartinov@yahoo.com (J.M.N.); loliciva@gmail.com (I.L.); pduschan@gmail.com (D.D.P.); 2 Faculty of Medicine, University of Belgrade, 11000 Belgrade, Serbia; mrj.stojanovic@gmail.com (M.S.); marko.aleksich@gmail.com (M.A.)

**Keywords:** liver cirrhosis, esophageal varices, variceal bleeding, non-invasive scoring systems

## Abstract

*Background and Objectives*: Upper endoscopy is considered the gold standard for screening and diagnosis of esophageal varices (EV). Non-invasive methods for predicting EV have become a research hotspot in recent years. The aim of this study was to assess the role of non-invasive scores in predicting the presence of EV in patients with liver cirrhosis, and to determine the value of these scores in predicting the outcome of patients with cirrhosis presenting with acute variceal bleeding. *Materials and Methods*: A total of 386 patients with liver cirrhosis were included. The model for end-stage liver disease (MELD), aspartate aminotransferase (AST) to alanine aminotransferase (ALT) ratio (AST/ALT), AST to platelet ratio index (APRI), fibrosis-4-index (FIB-4), fibrosis index (FI), King’s Score, albumin-bilirubin (ALBI) score, and platelet-albumin-bilirubin (PALBI) score were calculated. The discriminatory capacities of the examined scores in predicting the presence of esophageal varices were tested using receiver operating characteristic (ROC) curves. *Results*: The ROC curve analysis showed (area under the curve) AUC values of ALBI and PALBI of 0.603, and 0.606, respectively, for the prediction of EV. APRI, MELD, PALBI, King’s, FIB-4, and ALBI scores showed statistically significant correlation with EV bleeding (*p* < 0.05). AUC of APRI and MELD for predicting EV bleeding were 0.662 and 0.637, respectively. The AUC value of MELD in short-term mortality was 0.761. *Conclusions*: ALBI and PALBI scores had modest diagnostic accuracy of EVs in liver cirrhosis. APRI and MELD can be used as a reference index for the EV bleeding, and MELD score is best associated with short-term outcome in cirrhotic patients.

## 1. Introduction

Liver cirrhosis is a chronic disease characterized by hepatocyte necrosis, formation of regenerative nodules, and fibrosis of the liver tissue. In the majority of patients, as a result of these complex processes, portal hypertension develops. A hepatic venous pressure gradient (HVPG) measurement is the “gold standard’’ for the evaluation of the presence and severity of portal hypertension. Patients with cirrhosis may have either subclinical portal hypertension (PH) (HVPG is limited to 6–10 mmHg) or clinically significant PH (CSPH) (HVPG > 10 mmHg), which is further classified as severe (HVPG > 12 mmHg) and very severe PH (HVPG > 16 mmHg). In advanced cirrhosis, PH results in a profound hemodynamic derangement, which, in turn, leads to marked splanchnic vasodilation, and usually high HPVG > 16 mmHg [1].

HVPG is a robust surrogate marker in many clinical applications, such as diagnosis, risk stratification, identification of patients with hepatocellular carcinoma who are candidates for liver resection, monitoring the efficacy of medical treatment, and assessment of the progression of PH. However, this measurement is only possible in specialized centers. Additionally, the invasive nature of the procedure and an occasional need for repetition, bear the risk of possible complications. These limitations have contributed to the development of alternative methods of assessing PH severity. Several biochemical tests and serum concentrations of inflammatory biomarkers, as well as imaging techniques, have been reported to correlate with CSPH [2]. Adequate diagnosis of PH is important, however early diagnosis of subclinical PH is even more important, because of the preprimary prophylaxes that include etiotropic and pathogenetic treatment.

Although some manifestations of PH are clinically apparent (e.g., ascites), others, such as esophageal varices, may be silent until bleeding from them occurs. Approximately 50% of patients with cirrhosis develop esophageal varices over the course of the disease. The incidence rate of bleeding from untreated esophageal varices ranges from 20 to 76% [3,4,5]. Therefore, endoscopic screening is obligatory. High mortality rates of cirrhotic patients are in direct correlation with bleeding episodes from ruptured esophageal varices [6].

To date, upper gastrointestinal endoscopy remains the “gold standard’’ for diagnosing esophageal varices (EV), with Baveno VI Meeting Consensus recommending endoscopy screening for all cirrhotic patients at the time of diagnosis and periodical endoscopy examination in patients with EV [7]. However, upper endoscopy is an invasive procedure, which is unpleasant for the patient, and may not be cost-effective, considering the need for repeated interventions as well as the number of patients with suspected EV [8]. In recent decades, several non-invasive tests suggesting the presence of EV or bleeding from EV have been developed. Recent studies have showed that non-invasive scores, such as the model for end-stage liver disease (MELD), aspartate aminotransferase (AST) to alanine aminotransferase (ALT) ratio (AST/ALT), AST to platelet ratio index (APRI), fibrosis-4-index (FIB-4), fibrosis index (FI), and King’s score, are simple, non-invasive methods which could be used in the prediction of EV in cirrhotic patients [9,10,11,12,13,14,15,16]. Other predictive scores: age–bilirubin–INR–creatinine (ABIC) score, Glasgow alcoholic hepatitis score (GAHS), and Lille model, are useful in predicting the outcome of patients with alcoholic hepatitis [17]. The ABIC score was developed to categorize patients with AH into high-, moderate-, and low-risk groups based on the risk of death at 90 days and 1 year [18]. The Lille score evaluates the response in serum bilirubin after a 7-day course of corticosteroid therapy and aids the decision to either stop the corticosteroids or complete a 28-day course [19].

The Child–Turcotte–Pugh (CTP) and MELD scores are two of the most commonly used scores in everyday practice for patients with liver cirrhosis. Johnson et al. [20] developed the albumin-bilirubin (ALBI) score derived from two laboratory variables, bilirubin and albumin, without using factors evaluated subjectively (such as ascites and encephalopathy) or otherwise obtained [20]. In recent years, Roayaie et al. [21] proposed modifying the ALBI score by incorporating platelet count into ALBI in order to measure of liver function reserve. The PALBI model has recently been used as a predictor of mortality in patients with cirrhosis related complications [22]. Bearing in mind the numerous non-invasive scores used in patients with liver cirrhosis, our aim was to determine which has the highest diagnostic accuracy in predicting the presence of EV in patients with liver cirrhosis, as well as to determine the value of these scores in predicting the outcome of patients with cirrhosis presenting with acute variceal bleeding.

## 2. Materials and Methods

We conducted a retrospective single-center study in the Emergency Center of the University Clinical Center of Serbia. Our study included 386 patients who were admitted to our hospital between 2007 and 2021. The inclusion criteria were: (1) patients diagnosed with decompensated liver cirrhosis, based on clinical data, laboratory tests, liver imaging and/or histological report; (2) patients underwent both laboratory tests, ultrasonography and endoscopic examinations. The exclusion criteria were: (1) age under 18 years; (2) diagnosis of malignant tumor; (3) prior hepatic operation or splenectomy; (4) prior transjugular intrahepatic portosystemic shunt; (5) thrombosis of any part of the portal venous system; (6) current or previous history of lympho-proliferative diseases. The study was in accordance with the regulations of The Ethic Committee of our institution (Approval number: 602/2). The study was conducted according to the principles of the Helsinki Declaration (1989). Written informed consent was obtained from patients included in the study.

### 2.1. Data Collection

We collected the following data from electronic medical records: age, sex, etiology of liver diseases, ascites, hepatic encephalopathy (HE), history of upper gastrointestinal bleeding (UGIB), endoscopic findings, red blood cells (RBC), hemoglobin (Hb), white blood cells (WBC), platelets (PLT), alanine aminotransferase (ALT), aspartate aminotransferase (AST), alkaline phosphatase (ALP), γ-glutamine transferase (GGT), international normalized ratio (INR), albumin (ALB), total bilirubin (TBIL), blood urea nitrogen (BUN), and creatinine (Cr). Additionally, we calculated the model for end-stage liver disease (MELD) [23], APRI [24], AAR [25], FIB-4 [26], FI [27], King’s score [28], Lok index [29], ABIC [17], ALBI [20], and PALBI [21]. PALBI was categorized as: PALBI 1 (score ≤ 2.53), PALBI 2 (score > 2.53 and ≤2.09), and PALBI 3 (score > 2.09) [16]. All of the scores are presented at Table 1.

### 2.2. Evaluation of EVs

Each patient underwent upper endoscopy. The severity of EVs was classified as none, mild, and moderate/severe, based on the Baveno VI consensus and the American Association for the Study of Liver Disease (AASLD) practice guidelines [30]. Linear formation of EV was considered as mild, snake-like formation considered as moderate, while bead-like or tubercular formation was considered as severe. The diagnosis of variceal hemorrhage was established when active bleeding from an esophageal or gastric varicose vein was observed, or when a sign of recent bleeding, such as red cherry spots was observed.

### 2.3. Statistical Analysis

Statistical analysis was performed with SPSS ver. 20.0 (IBM, Chicago, IL, USA) (Student’s *t* test, Mann–Whitney test, chi square test). Demographic and clinical characteristics were presented by basic descriptive statistics, including means, medians, interquartile range (IQR), standard deviations, ranges, and percentages. The Kolmogorov–Smirnov test was used for examining the normality of distribution. Sensitivity and specificity, as well as the best cut-off value for the diagnosis of EV, were calculated using receiver operating characteristic (ROC) curves, with the Youden’s index for determining the best cut-off values. Correlation was examined using Pearson’s and Spearman’s correlation test. A *p* value less than 0.05 was considered statistically significant.

## 3. Results

### 3.1. Demographic Characteristics

We evaluated retrospectively 386 patients with liver cirrhosis who met the inclusion criteria. Baseline clinical characteristics and laboratory data of all patients are shown in Table 2.

Patients with EV were dominantly male, with significantly different values of platelets, total bilirubin, albumin, AST, ALP, GGT, LDH, Na, D-dimer, triglycerides, MELD, AAR, FIB-4, ALBI, and PALBI score, when compared to patients without EV (Table 3). Regarding the etiology of cirrhosis, a statistically significant difference was found in the EVs group (*p* < 0.05). Namely, patients with alcoholic hepatitis, HCV and cryptogenic liver cirrhosis, had a statistically significant (*p* < 0.05) presence of EV when compared to other etiologies (*p* = 0.010 vs. 0.033 vs. 0.040, respectively). (Table 4).

### 3.2. Non-Invasive Scores in Prediction of EV

**Figure 1:**
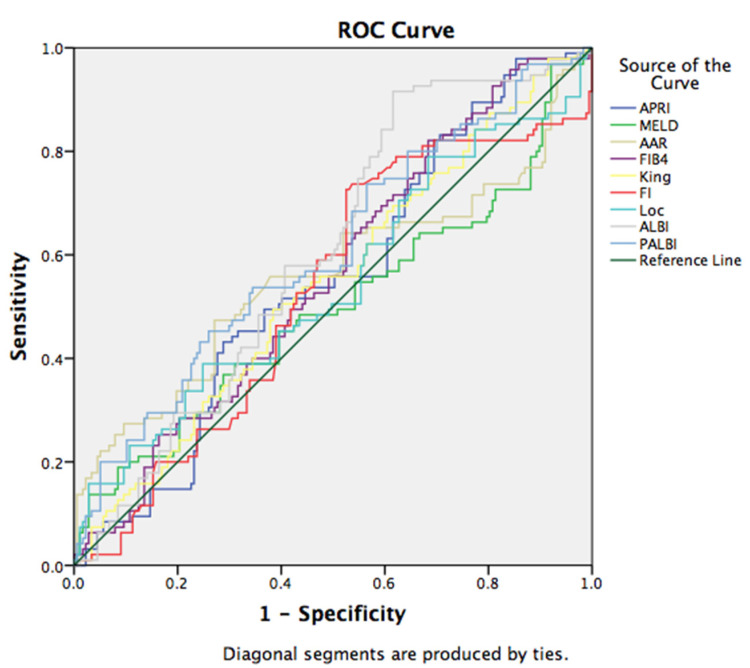
Receiver operating characteristic (ROC) curve for the discriminative ability of the prognostic scores to detect the presence of esophageal varices.

We found a statistically significant correlation between the ALBI and PALBI scores and the presence of EV in the study group (*p* < 0.05). The discriminatory capabilities of the examined scores in predicting the presence of EV were tested using ROC curves. ALBI had the discriminative ability to predict the presence of esophageal varices with the sensitivity of 74.7%, and specificity of 45.2% for the cut-off value −1.43 (area under curve (AUC): 0.603, 95% CI (0.535, 0.671)). Similar sensitivity (73.7%) was observed in the PALBI score, with specificity of 43.5% for the cut-off value of −1.79 (AUC: 0.606, 95% CI (0.535, 0.677)) (Figure 1).

### 3.3. Non-Invasive Scores in Prediction of EV Bleeding

Additionally, our results demonstrated that on admission, 142 (37.17%) patients experienced EV bleeding. Among the examined non-invasive tests, APRI, MELD, King’s score, as well as ALBI and PALBI, were statistically significantly correlated with EV bleeding (*p* < 0.05) (Table 5).

**Figure 2:**
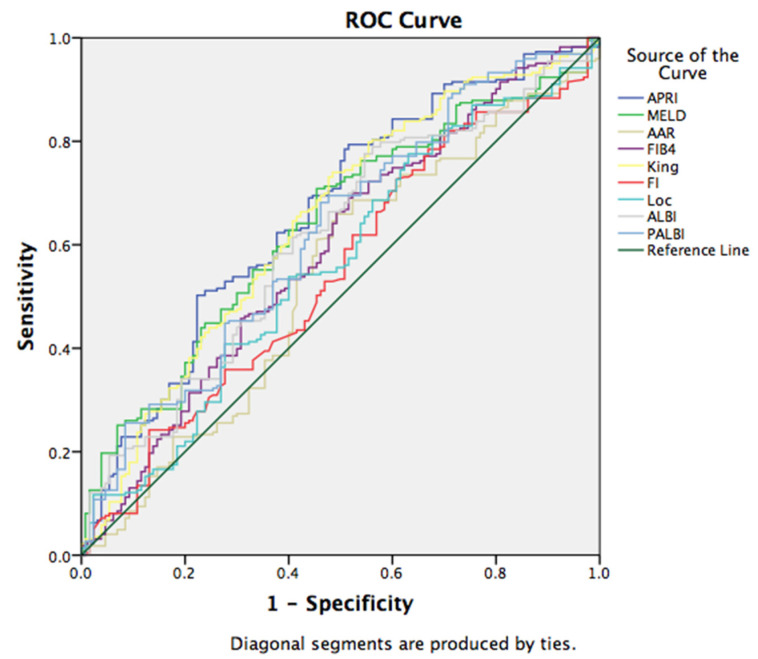
ROC curve for the discriminative ability of the prognostic scores to detect the presence of variceal bleeding in patients with liver cirrhosis.

For the assessment of EV bleeding, ROC curve analysis showed a sensitivity of 70.1% and specificity of 49% for the cut-off value of 1.36 for APRI (AUC: 0.662, 95% CI (0.603, 0.721)); sensitivity of 70.1% and specificity of 69% for the cut-off value of 18 for MELD (AUC: 0.637, 95% CI (0.578, 0.696)), with other scores presented in Figure 2. Furthermore, a statistically significant association was found between serum sodium values and bleeding (*p* = 0.000), where low sodium values were frequently seen in patients with EV bleeding (*p* = 0.001, *p* < 0.05) (Table 6).

### 3.4. Non-Invasive Scores in Prediction of Short Term Mortality

**Figure 3:**
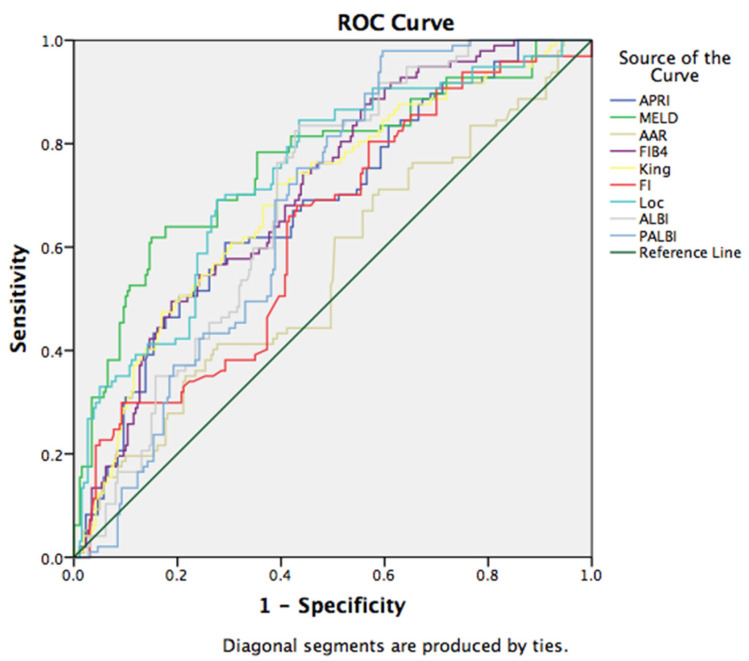
ROC curve for the discriminative ability of the prognostic scores to detect short-term mortality.

Moreover, we have investigated a possible correlation between non-invasive scores and short-term mortality. All examined prognostic scores statistically significant correlated with mortality, and the MELD score showed the best predictive ability, with sensitivity of 73.2% and specificity of 65% for the cut-off value of 17, (AUC: 0.761, 95%CI (0.702, 0.8210, *p* = 0.000). The other non-invasive scores (Lok, FIB-4, King’s, ALBI, APRI, PALBI, FI, and AAR) have shown statistically significant correlation to mortality. However, we have not found a correlation of the ABIC score with mortality. Additional ROC curves have been provided in Figure 3.

## 4. Discussion

Considering that EV are one of the most common and potentially life-threatening complications of liver cirrhosis, there is a need for the identification of non-invasive markers for fast orientation in EV detection, and further risk and treatment assessment [14]. Taking into account the high cost of repeated upper endoscopies, especially in resource limited settings, simple non-invasive scores could potentially be of great interest in the daily clinical practice of developing countries.

Several previous studies evaluated the performance of multiple scoring systems in predicting EV, as well as variceal bleeding and mortality in patients with liver cirrhosis [14]. In the present study, we investigated the prognostic performance of all the available scores, relying solely on objective laboratory parameters, including MELD, AAR, APRI, FIB-4, FI, King’s, Lok, ABIC, ALBI, and PALBI scores, in predicting the presence of EV, variceal bleeding, and mortality in patients with liver cirrhosis.

The results of our analyses showed that MELD, ALBI, PALBI, AAR, and FIB-4 scores significantly correlated with the presence of EV, which is a result consistent with previous studies [31,32]. Specifically, the MELD score in the group of patients with EV was significantly higher compared to patients without EV, which is a result similar to the result of Kraja et al. [31]. Yun-Cheng Hsieh et al. [33] found that the ALBI score significantly correlated with HVPG and other hemodynamic parameters, with a higher correlation coefficient compared to that for other fibrosis markers, suggesting it potentially has a very important role in screening patients with advanced portal hypertension. The results of our study are consistent with the previous findings relating to the ALBI score and correlation to EV.

In the previously mentioned study of Kraja et al., FIB-4 was shown to be a significant predictor of EV presence [31]. However, Deng et al. demonstrated that FIB-4, as well as AAR, has moderate diagnostic accuracy in predicting EV, which are results consistent with our study [14].

We did not find data on the predictive power of ABIC, GAHS, or Lille score, in relation to the presence of esophageal varices in patients with liver cirrhosis. The results of our study showed that the ABIC score did not correlate with the presence of EV, bleeding from EV, nor outcome.

When we analyzed the association of non-invasive scores in the prediction of variceal bleeding, we found that there was a statistically significant association of APRI, MELD, and King’s score, as well as ALBI and PALBI, with bleeding. Similar to the findings of Oikonomou et al. [34], we found a significant correlation between ALBI and PALBI scores, and bleeding.

The present study evaluated the performance of the non-invasive scores in predicting in-hospital mortality after variceal bleeding. Among our patients, MELD, ALBI, and PALBI showed a highly statistically significant association with poor outcome. Zou et al. [35] previously tested the performance of the ALBI score in predicting in-hospital mortality of acute upper gastrointestinal bleeding, where their results showed a similar sensitivity of the MELD score, especially in patients treated with endoscopic therapy. As concluded by Elshaarawy et al., if the PALBI score could replace the CTP score and the MELD, it would be easier and quicker to identify candidates for an early transjugular intrahepatic porto-systemic shunt procedure, and it is also not subject to the inconsistencies of CTP, resulting from the inclusion of ascites and encephalopathy [32]. The performance of PALBI in predicting mortality was significantly better than the ALBI score, possibly because the PALBI score includes a platelet count, which reflects the effect of portal hypertension—the main underlying cause of acute variceal bleeding.

## 5. Limitation of the Study

Our study had some limitations. The study participants were cirrhotic patients with different etiologies of decompensated liver cirrhosis. Secondly, we did not separate patients into groups according to the degree of ALBI and PALBI scores. Thirdly, long-term follow-up was unavailable in our conditions. Therefore, this study could not evaluate the role of these non-invasive scoring systems for predicting the long-term prognosis. Fourthly, not all patients underwent fibroscan examination and, therefore, we did not present these data.

Despite the limitation of being a retrospective single-center study, all our patients underwent endoscopy, and experienced bleeding confirmed to be from varices, providing further validity to our findings.

## 6. Conclusions

In conclusion, our results suggest that ALBI and MELD scores are reliable predictors of esophageal varices in liver cirrhosis patients. Contrary to other findings, APRI, MELD, and King’s scores can be good options for predicting variceal bleeding among patients with liver cirrhosis, while MELD, ALBI, and PALBI scores have strong associations with in-hospital mortality after variceal bleeding. Although the MELD score has been used for years, it proved to be the only score among all those tested, which showed a significant correlation with all three examined variables in our study. It is an old non-invasive score, but could be useful as an initial screening tool for cirrhotic patients in areas which lack endoscopy facilities. The use of non-invasive scores for diagnosis of subclinical portal hypertension should be considered in future studies regarding patients with liver cirrhosis.

## Figures and Tables

**Table 1:** Calculation of non-invasive scores.

MELD score = 9.57 × ln(Cr) + 3.78 × ln(TBIL) + 11.2 × ln (INR) + 6.43
APRI = ((AST/UNL) × 100)/PLT
AAR = AST/ALT
FIB-4 = (age × AST)/PLT × ALT^1/2^
FI = 8 − 0.01 × PLT-ALB
King = age × AST × INR/PLT LogOddsLok = (1.26 × AST/ALT) + (5.27 × INR) − (0.0089 × PLT) − 5.56
Lok index = e^(LogOddsLok)^/(1 + e^(LogOddsLok)^)
ALBI = ((log10 bilirubin × 0.66) + (albumin × (−0.085)))
PALBI = 2.02 × log10 bilirubin − 0.37 × (log10 bilirubin)^2^ − 0.04 × albumin − 3.48 × log10 platelets + 1.01 × (log10 platelets)^2^
ABIC = (age × 0.1) + (serum bilirubin × 0.08) + (serum creatinine × 0.3) + (INR × 0.8)

Abbreviations: MELD—Model for end-stage liver disease, TBIL—Total serum bilirubin, APRI—AST to platelet ratio index, AST—aspartate aminotransferase, UNL—upper normal limit, PLT—platelets, ALT—alanine aminotransferase, AAR—aspartate aminotransferase-to-alanine aminotransferase ratio, FIB-4—fibrosis-4 index, FI—fibrosis index, ALBI—albumin-bilirubin, PALBI—platelet-albumin-bilirubin, ABIC—Age, bilirubin, INR, creatinine score.

**Table 2:** Clinical characteristics and laboratory data in patients with liver cirrhosis.

Variables	Total Patients (*n* = 386)
Sex (m/f)	309/77
Age (years)	62.4 ± 13.14
Etiology, *n* (%)	
Alcohol	273 (70.7)
Hepatitis B virus	17 (4.4)
Hepatitis C virus	18 (4.7)
Autoimmune disease	26 (6.7)
Wilson disease	3 (0.8)
Toxic	3 (0.8)
Cryptogenic	52 (13.5)
Laboratory test	
Hb (g/L) ^a^	111.10 ± 81.62
WBC (10^9^/L) ^a^	9.35 ± 5.23
PLT (10^9^/L) ^a^	129.887 ± 85.65
TBIL (mmol/L) ^b^	81 (100)
Alb (g/L) ^b^	27 (5)
AST (U/L) ^b^	82 (97)
ALT (U/L) ^b^	31 (27)
ALP (U/L) ^b^	156 (213)
GGT (U/L) ^b^	68 (1590)
BUN (mmol/ L) ^b^	13.2 (25)
Cr (µmol/L) ^b^	115 (268)
INR ^b^	1.7 (1.78)
D-dimer (mg/L) ^b^	6.18 (28.1)
CRP (mg/L) ^b^	43.5 (127)
Pct (ng/L) ^b^	3.0 (12.7)
Na (mmol/L) ^b^	136.20 ± 6.14
K (mmol/L) ^b^	4.28 ± 0.92
LDH (U/L) ^b^	675 (1168)
NH4 (µmol/L) ^b^	89 (59)
Cholesterol (mmol/L)	3.30 ± 1.97
Triglycerides (mmol/L)	1.27 ± 0.82
MELD score ^b^	16.4 (13.5)
APRI score ^b^	1.48 (2.64)
AAR score ^b^	1.9 (1.12)
FIB-4 score ^b^	6.15 (7.66)
King score ^b^	1036 (1841.28)
FI score ^b^	4.1 (1.33)
Loc score ^b^	4.08 (3.97)
ALBI score ^b^	−1.11 (0.89)
PALBI score ^b^	−1.54 (0.66)
ABIC score ^b^	8.43 (2.15)

Abbreviations: ^a^ mean ± SD, ^b^ median(IQR), pts—patients, *n*—number of patients, Hb—hemoglobin, WBC—white blood cell, Plt—platelet, TBil—total bilirubin, Alb—albumin, AST—aspartate aminotransferase, ALT—alanine aminotransferase, ALP—alkaline phosphatase, GGT—gamma glutamyltransferase, BUN—blood urea nitrogen, Cr—creatinine, INR—international normalized ratio, CRP—C-reactive protein, Pct—procalcitonin, Na—sodium, K—potassium, LDH—lactic acid dehydrogenase, NH4—ammonium ion, MELD—model for end-stage liver disease, APRI—AST to platelet ratio index, AAR—aspartate aminotransferase-to-alanine aminotransferase ratio, FIB—fibrosis-4 index, FI—fibrosis index, ALBI—albumin-bilirubin, PALBI—platelet-albumin-bilirubin, ABIC—Age, bilirubin, INR, creatinine score.

**Table 3:** Values of laboratory analysis and scores in relation to the presence of esophageal varices.

Variables	With Varices Pts	Without Varices Pts	*p* Value
Hb (g/L) ^a^	114.61 ± 113.93	102.67 ± 23.45	0.041
WBC (10^9^/L) ^a^	9.715 ±5.52	9.612 ± 5.16	0.785
Plt (10^9^/L) ^a^	124.59± 65.95	142.51 ± 120.02	0.000
TBil (mmol/L) ^b^	37.6	48.6	0.009
Alb (g/L) ^b^	29.0	27.0	0.028
AST (U/L) ^b^	47.0	66.0	0.006
ALT (U/L) ^b^	27.0	28.5	0.238
ALP (U/L) ^b^	88.0	110.5	0.024
GGT (U/L) ^b^	77.0	82.5	0.010
BUN (mmol/L) ^b^	10.3	8.4	0.184
Cr (µmol/L) ^b^	81.0	73.0	0.009
INR ^b^	1.49	1.48	0.965
D-dimer (mg/L) ^b^	3.45	5.57	0.007
CRP (mg/L) ^b^	14.85	30.9	0.056
Pct (ng/L) ^b^	0.57	1.32	0.282
Na (mmol/L)	136.76 ± 5.92	136.02 ± 6.75	0.005
LDH (U/L) ^b^	451.0	591.5	0.000
NH4 (µmol/L) ^b^	59.0	81.0	0.091
Cholesterol (mmol/L)	3.238 ± 1.30	2.717 ± 1.31	0.699
Triglycerides (mmol/L)	1.145 ± 0.52	1.382 ± 1.17	0.002
MELD score ^b^	14.79	16.53	0.049
APRI score ^b^	1.30	1.51	0.132
AAR score ^b^	1.78	2.08	0.037
FIB-4 score ^b^	5.55	6.91	0.047
King score ^b^	887.9	1085.7	0.171
ALBI score ^b^	−1.29	−1.12	0.002
PALBI score ^b^	−1.67	−1.52	0.005
ABIC score ^b^	7.93	8.59	0.815

Abbreviations: ^a^ mean ± SD, ^b^ median(IQR), pts—patients, *p*—probability value, Hb—hemoglobin, WBC—white blood cell, Plt—platelet, TBil—total bilirubin, Alb—albumin, AST—aspartate aminotransferase, ALT—alanine aminotransferase, ALP—alkaline phosphatase, GGT—gamma glutamyltransferase, BUN—blood urea nitrogen, Cr—creatinine, INR—international normalized ratio, CRP—C-reactive protein, Pct—procalcitonin, Na—sodium, K—potassium, LDH—lactic acid dehydrogenase, NH4—ammonium ion, MELD—model for end-stage liver disease, APRI—AST to platelet ratio index, AAR—aspartate aminotransferase-to-alanine aminotransferase ratio, FIB-4—fibrosis-4 index, FI—fibrosis index, ALBI—albumin-bilirubin, PALBI—platelet-albumin-bilirubin, ABIC—Age, bilirubin, INR, creatinine score.

**Table 4:** The presence of esophageal varices in relation to the etiology of cirrhosis.

Etiology	Pts with Varices (%)	Pts without Varices (%)	*p*
Alcohol	118 (59.0%)	82 (41.0)	0.010
HBV cirrhosis	10 (76.9)	3 (23.1)	0.242
HCV cirrhosis	14 (87.5)	2 (12.5)	0.033
Autoimmune disease	16 (69.6)	7 (30.4)	0.359
Wilson disease	2 (100)	0 (0)	0.406
Toxic disease	0 (0)	1 (100)	0.362
Cryptogenic disease	35 (76.1)	11 (23.9)	0.040

Abbreviations: pts—patients, p—probability value, HBV—hepatitis B virus, HCV—hepatitis C virus.

**Table 5:** Correlation between the scores and the presence of variceal bleeding in patients with liver cirrhosis.

Scores	Pts with Variceal Bleeding	Pts without Variceal Bleeding	*p*
APRI	3.35	1.87	0.000
MELD	18.95	17.15	0.000
AAR	3.12	1.92	0.887
FIB-4	13.75	6.83	0.109
King	1289.39	1179.46	0.000
ALBI	−1.00	−1.09	0.027
PALBI	−1.48	−1.52	0.000
ABIC	8.39	8.49	0.627

Abbreviations: pts—patients, *p*—probability value, APRI—AST to platelet ratio index, MELD—model for end-stage liver disease, AAR—aspartate aminotransferase-to-alanine aminotransferase ratio, FIB-4—fibrosis-4 index, ALBI—albumin-bilirubin, PALBI—platelet-albumin-bilirubin.

**Table 6:** Correlation between serum sodium value and variceal bleeding.

		B	S.E.	Wald	df	Sig.	Exp (B)	95% CI for EXP (B)	
								Lower	Upper
Step 1	Na Constant	0.111 −15.756	0.022 3.031	25.269 27.032	1 1	0.000 0.000	1.117 0.000	1.070	1.167

Abbreviations: Na—Sodium.

## Data Availability

All data are available from corresponding authors upon request.
